# Modeling the Embrace of a Mutator: APOBEC Selection of Nucleic Acid Ligands

**DOI:** 10.1016/j.tibs.2018.04.013

**Published:** 2018-08

**Authors:** Jason D. Salter, Harold C. Smith

**Affiliations:** 1 OyaGen, Inc., 77 Ridgeland Road, Rochester, NY 14623, USA; 2 University of Rochester, School of Medicine and Dentistry, Department of Biochemistry and Biophysics, 601 Elmwood Avenue, Rochester, NY 14642, USA

**Keywords:** APOBEC, cancer, crystal structure, cytidine deaminase, gene editing, innate and adaptive immunity, mutation, RNA and DNA binding, RNA editing, structural biology, zinc-dependent deaminase.

## Abstract

The 11-member APOBEC (apolipoprotein B mRNA editing catalytic polypeptide-like) family of zinc-dependent cytidine deaminases bind to RNA and single-stranded DNA (ssDNA) and, in specific contexts, modify select (deoxy)cytidines to (deoxy)uridines. In this review, we describe advances made through high-resolution co-crystal structures of APOBECs bound to mono- or oligonucleotides that reveal potential substrate-specific binding sites at the active site and non-sequence-specific nucleic acid binding sites distal to the active site. We also discuss the effect of APOBEC oligomerization on functionality. Future structural studies will need to address how ssDNA binding away from the active site may enhance catalysis and the mechanism by which RNA binding may modulate catalytic activity on ssDNA.

## Highlights

APOBEC proteins catalyze deamination of cytidine or deoxycytidine in either a sequence-specific or semi-specific manner on either DNA or RNA.

APOBECs each possess the cytidine deaminase core fold, but sequence and structural differences among loops surrounding the zinc-dependent active site impart differences in sequence-dependent target preferences, binding affinity, catalytic rate, and regulation of substrate access to the active site among the 11 family members.

APOBECs also regulate the deamination reaction through additional nucleic acid substrate binding sites located within surface grooves or patches of positive electrostatic potential that are distal to the active site but may do so nonspecifically.

Binding of nonsubstrate RNA and RNA-mediated oligomerization by APOBECs that deaminate ssDNA downregulates catalytic activity but also controls APOBEC subcellular or virion localization.

The presence of a second, though noncatalytic, cytidine deaminase domain for some APOBECs and the ability of some APOBECs to oligomerize add additional molecular surfaces for positive or negative regulation of catalysis through nucleic acid binding.

## APOBEC Interactions with Nucleic Acid Substrate are Critical to Understanding Physiological Function

Each member of the APOBEC family has a specific set of physiological functions that involve binding of nucleic acid and catalysis of **cytidine to uridine deamination** (see Glossary) in context of either RNA and/or single-stranded DNA (ssDNA) [1]. Cytidine deamination by specific APOBEC proteins has well-regulated, physiological roles in restriction of endogenous and exogenous retroviruses, innate and adaptive immunity, epigenetics, and lipid metabolism. (Box 1, reviewed in 2, 3). The molecular basis for all APOBEC functions requires direct interactions with nucleic acids. Understanding the molecular basis of these interactions is critical to understanding the basis for diseases that occur upon misregulation. APOBEC deaminase activity is regulated by their subcellular distribution, their expression level, and in some cases, binding of a protein cofactor or nonsubstrate RNA. However, off-target mRNA and gene editing have been documented throughout the history of this field and have been implicated in numerous cancers (recently reviewed in 4, 5, 6, 7, 8). To understand the circumstances and mechanisms that determine APOBEC/AID editing site fidelity or off-target mutagenesis, this review will focus on the emerging understanding of the molecular and structural requirements of APOBEC interactions with both **substrate and nonsubstrate nucleic acids**. **Co-crystal structures** of APOBEC proteins with bound RNA or DNA mono- or oligonucleotides and their corresponding accession numbers, resolution, and contributors are listed in Table 1.

**Table 1:** Select Structures of APOBECs Bound to Either Nucleic Acid or Nucleotides

APOBEC	Species	Ligand sequence (modeled)	PDB ID	Resolution (Å)	Study	Refs
A3A	Human	5′-dTdTdCdTdT-3′	5KEG	2.2	Kouno *et al*. 2017	[76]
A3A	Human	5′-dAdTdCdGdGdG-3′	5SWW	3.15	Shi *et al*. 2017	[77]
A3B-(Loop1-A3A) chimera	Human	5′-dTdTdCdA-3′	5TD5	1.72	Shi *et al*. 2017	[86]
A3B	Human	dCMP	5CQH	1.73	Shi *et al*. 2015	[60]
A3F	Human	Poly-dT_10_	5W2M	3.7	Fang *et al*. 2018	[87]
A3G (N)	Rhesus macaque	Poly-dT_10_	5K83	2.39	Xiao *et al*. 2016	[69]
A3H	Human	Endogenous RNA (8-mer mixed sequence)	6BOB	3.43	Shaban *et al*. 2018	[115]
A3H	Pig-tailed macaque	Endogenous RNA (9-mer mixed sequence)	5W3V	2.24	Bohn *et al*. 2017	[114]
AID	Human	dCMP	5W0U	3.61	Qiao *et al*. 2017	[95]

Box 1 **Diverse Cellular Functions Affected by APOBEC Proteins**

A1 was first characterized for its RNA editing of a specific cytidine (C6666) of *apoB* mRNA, which encodes a truncated form of the ApoB protein (reviewed in [2]). Both the truncated and full-length variants of ApoB protein bind to lipids and cholesterol. Cholesterol transport in the blood with the full-length protein is associated with an increased risk of atherosclerosis and as such, editing of the *apoB* mRNA may mitigate this risk. Neurofibromin mRNA also is site-specifically edited and produces a truncated protein, lacking its tumor suppressor function [9]. In addition, there are potentially numerous A1-dependent C to U editing sites within 3′ untranslated regions of a variety of mRNAs [10]; editing at these sites may alter mRNA stability. A1 editing requires a cis-acting mooring sequence motif within substrate mRNAs and is the only APOBEC known to require an RNA-binding protein cofactor, either A1 complementation factor (A1CF) [11] or RBM47 [12], for substrate targeting.

AID is imported into the nucleus of activated germinal center B cells, where it mutates the immunoglobulin gene locus through multiple dC to dU deaminations. This leads to **hypermutation** of the immunoglobulin variable region [somatic hypermutation (SHM)] and thus, enables diversification of the immunoglobulin variable region [13]. Hypermutation of the immunoglobulin constant region produces antibodies with a variety of effector functions by inducing either class switch recombination (CSR) or gene conversion (GC) [14]. AID editing of dC to dU occurs on single-stranded immunoglobulin genes during transcription in greatest frequency within 5′-dWdRdC-3′, known as a hotspot motif [15]. Base excision repair of dU produces either a variety of point mutations that may encode diversity in the amino acids within the variable region of immunoglobulins (SHM) or may prompt double-stranded breaks necessary for nonhomologous recombination of the constant region of immunoglobulins (CSR and GC) [16].

The seven A3 enzymes target dC in ssDNA in a variety of retroelements and retroviruses and prefer editing dC in the context of the dinucleotide sequence 5′-dTdC-3′, except for A3G which prefers 5′-dCdC-3′ 17, 18. A3D, A3F, A3G, and A3H haplotype II provide various levels of anti-HIV activity in T cells through cytidine deamination of single-stranded genomic cDNA during reverse transcription 19, 20; A3B, A3C, A3F, and A3G also prevent transmission of simian immunodeficiency virus to humans 21, 22, 23.

A3 enzyme-induced mutations are also implicated in inhibition of DNA viruses. A3B, A3C, A3D, A3G, and A3H are upregulated in hepatocytes by interferon-α and -γ 24, 25 or by expression of heat-shock proteins [26] and induce genomic mutations in hepatitis B virus. Conversely, A3A alone has been shown to inhibit the parvovirus, adeno-associated virus type 2 in a deaminase-independent manner 27, 28, 29, potentially due to structural differences of polynucleotide binding grooves near the catalytic site compared with other A3 members [30].

All A3s (A–H) inhibit the **retrotransposition** of the autonomous **long interspersed nuclear elements (LINE)** and the nonautonomous **short interspersed nuclear elements (SINE)** endogenous retroelements 31, 32. Inhibition of LINE and SINE retrotransposition may be through hypermutation of retroelement ssDNA [33] or through deaminase-independent mechanisms that may involve sequestering SINE RNAs as large ribonuclear protein complexes 34, 35.

Regulation of APOBEC activity is critical because during transcription, genomic ssDNA is susceptible to APOBEC-mediated mutation and subsequent progression of a variety of cancers 6, 36. In fact, the mutational signature of APOBECs (mutations of dC within a dTdC dinucleotide) is widespread among cancers and misregulation of the DNA-editing members of the family has been implicated in localized clusters of hypermutations 37, 38. Although the A3 family is mainly cytoplasmic, A3A does travel to the nucleus and A3B is predominantly localized to the nucleus [39], affording access to genomic DNA. While misregulated editing activities of A3A 40, 41, A3B [42], A3H [43], AID [44], and A1 [45] are specifically associated with cancer, as DNA modifying enzymes, a role for the other APOBECs in cancer cannot be ruled out.

In contrast, very little is known about the physiological targets or nucleic acid sequence preferences of A2 and A4, although A2 is known to be expressed in skeletal and cardiac muscle [46] and A2 gene knockout mice displayed mitochondrial defects [47].

Alt-text: Box 1

## The Canonical Cytidine Deaminase Fold of the APOBEC Family

**Figure 1:**
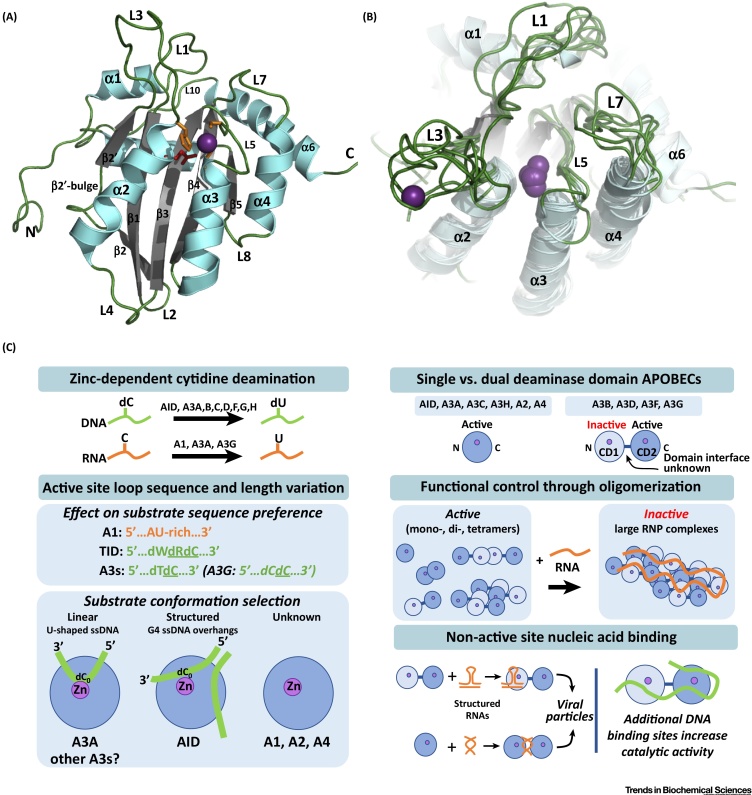
Key Figure: Variation of the Cytidine Deaminase Fold Drives Diversity of APOBEC Function

The APOBECs are members of the large cytidine deaminase superfamily that contain the canonical zinc-dependent deaminase (ZDD) signature motif (HxEx_25-30_PCx_2-4_C) embedded within the core cytidine deaminase fold. This fold comprises a five-stranded mixed β-sheet surrounded by six α-helices with the order α1-β1-β2-α2-β3-α3-β4-α4-β5-α5-α6 (Figure 1A, Key Figure).

Deamination of cytidine to uridine is a conserved concerted reaction requiring zinc (reviewed in [48]). It is catalyzed in a deep substrate-binding pocket located at the nexus of the N terminal ends of α2 and α3 helices by elements of the ZDD. The conserved His and Cys residues coordinate the catalytic zinc ion; a water molecule completes the quartet of zinc coordination and is thus activated for nucleophilic attack on the C4 atom of the cytidine ring. The conserved glutamic acid acts as a proton shuttle during proton transfer from the activated water to the leaving ammonia group, N3 of the cytidine base. This enzymatic mechanism is conserved among the cytidine deaminase family. All APOBECs except A2 and A4 deaminate dC-to-dU in the context of ssDNA or RNA. A1 was first described for C-to-U RNA editing (reviewed in 2, 3) but A1, A3A, and A3G have the ability to deaminate cytidines in both ssDNA and single-stranded RNA (ssRNA) 49, 50, 51, 52, 53.

APOBEC proteins comprise either one (A1, AID, A2, A3A, A3C, A3H, and A4) cytidine deaminase domain or two (A3B, A3D, A3G, and A3F) domains in tandem. The C terminal domain (CD2) of each of the dual-deaminase domain APOBECs is catalytically active while the N terminal domains (CD1) do not have the ability to deaminate substrate, even though they maintain the core cytidine deaminase fold. CD1 binding to ssDNA and RNA may up- or downregulate the catalytic activity of the adjacent CD2 but the molecular basis of this regulatory role is unknown. The tertiary structure orientation of CD1 and CD2 of the dual-deaminase domains and their interface remains an open question.

Though the core fold is maintained, subtle sequence differences among the APOBECs have led to differences in surface charge, active site interactions, and oligomeric propensity, as well as differences in loop length, configuration, and plasticity (recently reviewed in 1, 54). These differences are thought to have evolved as the family of present day proteins and enable the variety of functional characteristics for each.

Numerous crystallographic structures of APOBECs have been solved without nucleic acid or nucleotide ligands bound, including: (i) the single-domain APOBECs, A2 [55], AID [56], A3A [57], A3C [58], and A3H [59]; (ii) the C terminal catalytic domains (CD2) of the dual-domain APOBECs, A3B [60], A3G 61, 62, 63, 64, and A3F 65, 66; and (iii) the N terminal noncatalytic (CD1) domains of A3B [67] and A3G 68, 69. Likewise, solution NMR structures of unliganded APOBECs have been solved for A2 [70], A3A [71] and for the catalytic domains of A3B [72] and A3G 73, 74, 75. These structures, accompanied by structure-function based mutational assays, have provided insight into form and function of this family (reviewed in 1, 54). Amino acid sequence variability in loops L1, L3, L5, and L7 that surround the conserved active site pocket of catalytically active domains are integral to the diversity of substrate binding affinity, catalytic rate, active site occlusion, and dinucleotide nearest-neighbor sequence preference selection that is present in the APOBEC family (Figure 1B and reviewed in [1]). Variations of molecular surface properties on the conserved core cytidine deaminse fold provide additional nucleic acid binding sites that together drive the variety of oligomeric propensities and cellular localization. Many questions remain regarding structural specificity of APOBECs with their cognate nucleic acid substrates, both at and distal from the active site. The field has begun addressing these questions with co-crystal structures of APOBECs bound to nucleic acid ligands. The structure-function relationship of the APOBEC family that is driven by variation of the conserved cytidine deaminase fold is summarized in Figure 1C, which also serves as a guide for the topics discussed herein.

## An A3 ssDNA Substrate Has an Unexpected U-shaped Orientation

**Figure 2:**
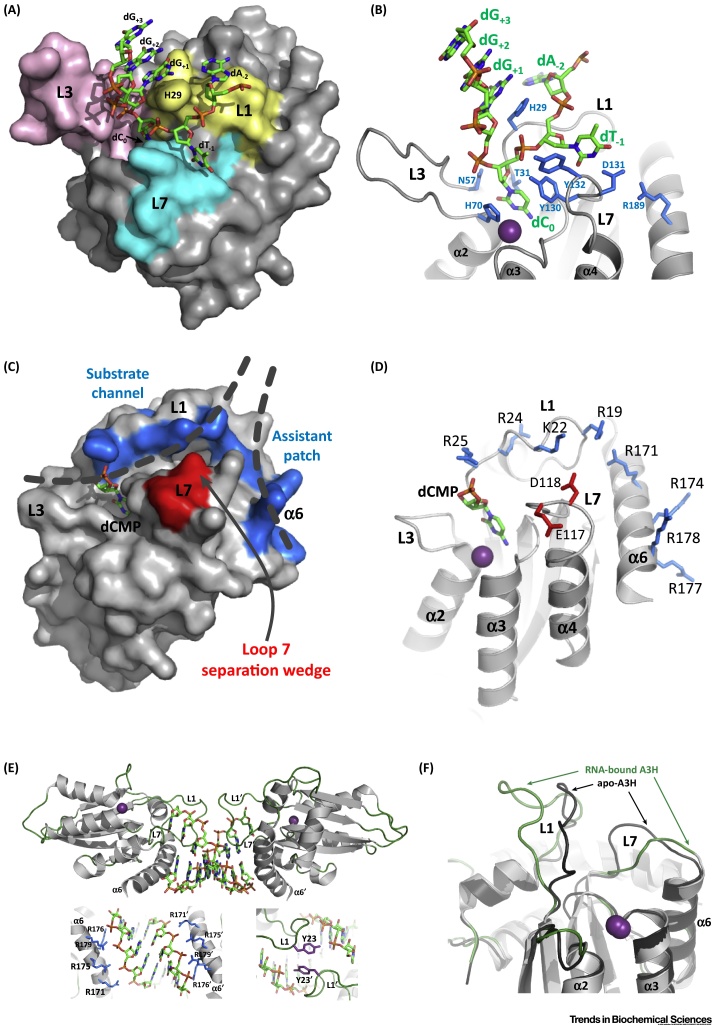
APOBEC Interactions with Substrate and Regulatory Nucleic Acid Differ Despite a Common Core. (A) The co-crystal structure of A3A (E72A) with linear ssDNA substrate showing 5′-dA_-2_dT_-1_dC_0_dG_+1_ dG_+2_dG_+3_-3′, where dC_0_ is substrate cytidine (PDB 5SWW, Table 1). A3A molecular surface is shown in gray, with surface residues of L1, L3, and L7 colored yellow, pink, and blue, respectively. DNA is shown in stick representation with atoms of carbon, phosphate, oxygen, and nitrogen colored green, orange, red, and blue, respectively. The DNA binds a surface groove between L1, L3, and L7 and takes on a tight U-shaped configuration centered on dC_0_, which is flipped out and buried in the active site pocket; a dT nucleotide is preferred at position –1 and it is shown buried in a shallow surface pocket of L7 residues. (B) A ribbon diagram representation of A3A illustrating sidechains (blue) of residues involved in critical binding interactions with ssDNA substrate or in maintaining competent binding site configuration. The catalytic zinc ion is shown as a purple sphere. (C) AID from the co-crystal structure with a dCMP ligand (PDB 5WOU, Table 1) is shown as a molecular surface and as a cartoon diagram (D) in the same orientation and illustrates regions of the bifurcated binding surface model for recognition of G4-structured DNA substrates. In both (C) and (D) the dCMP ligand is depicted with sticks and colored as in (A); the catalytic zinc ion is shown in (D) as a purple sphere. ssDNA overhangs 3′ of the G4 core structure are postulated to bind a substrate channel formed by positively charged L1 residues that are conserved in AID from zebra fish to humans. A second ssDNA of the branched nucleic acid structure is predicted to bind the ‘assistant patch’, a positively charged surface patch formed by conserved residues of α6-helix. These conserved negatively charged residues are depicted as blue surfaces (C) and sidechain sticks (D). Negatively charged residues of L7 serve as a separation wedge for the negatively charged ssDNA backbones and are depicted as a red surface (C) and sidechain sticks (D). In (C) dashed lines indicate the predicted path of ssDNA in the substrate channel and assistant patch, which diverge at the separation wedge. As with other APOBECs, L1, L3, L5, and L7 are clustered near the active site, but in contrast to other APOBECs, α6-helix in AID is believed to play a major role in substrate stabilization and conformation selection through conserved, positively charged residues. (E) The co-crystal structure of dimeric human A3H in complex with an 8-mer RNA duplex (PDB 6BOB, Table 1). A3H molecules are shown as cartoons with gray colored α-helices and β-strands and green loops. RNA molecules are depicted as sticks, with atom coloration as in (A). Each A3H molecule makes significant contact with the RNA molecules but does not form protein–protein interactions. Electrostatic interactions between RNA backbone and α6-helix arginines and L1 tyrosine are shown in the insets. (F) The same co-crystal structure from (E) with RNA removed for clarity is shown superposed with the crystal structure of human apoA3H (PDB 5W45) (dark gray loops). The conformation of L1 and 7 are distorted to accommodate RNA duplex compared with apoA3H.

The conformation and structural mechanism of ssDNA substrate binding were largely unknown until co-crystal structures of A3A with bound ssDNA substrate were recently solved with either three [76] or six [77] nucleotides of substrate DNA resolved (Figure 2A). Substrate DNA backbone bound within a deep U-shaped groove formed by L1, 3, 5, and 7 of A3A centered around H29 sidechain of Loop 1 with both dC_0_ and dT_-1_ pyrimidine ring flipped into protein pockets, where dC_0_ is defined as the cytidine that is deaminated (Figure 2B). The dC_0_ base was sandwiched between T31 and H70 sidechains and formed a T-shaped pi-stack with Y130 in the deep active site pocket. The amino (leaving) group at C4 was positioned proximal to the zinc-activated water by a bifurcated H-bond with carbonyl oxygens of W98 and S99. N57 also contributed stabilizing interactions with the dC_0_ backbone and sugar that supported proper orientation of the cytidine base in the active site.

The sidechain of H29 is predicted to have a major role in stabilizing the U-shaped conformation of the substrate and likely drives a ‘latch and release’ mechanism of A3A interaction with substrate. In the unliganded A3A structure the H29 sidechain rotamer is undefined [71]. Upon substrate binding it orients (the ‘latch’) to form extensive hydrogen bonds with the backbone and a stacking interaction with the base at position +1. Release of the deaminated product after catalysis is predicted to result from destabilization of interactions within the active site, resulting from an inability to maintain the hydrogen bonding network centered on H29 and the subsequent rearrangement of the H29 rotamer.

### A3 Family Preferences for Substrate Sequence Are Determined by Loop 1 and 7

Specificity of A3A for pyrimidine at the –1 position of its preferred substrate was born out by the extensive van der Waal contacts and hydrogen bonds between the Watson-Crick (WC) edge of dT_-1_ and residues Y130, D131, and Y132 of Loop 7 and W98 of Loop 5. The size of the –1 pocket in A3A accommodated similar bonding with the WC edge of a dC at –1 position but precluded the larger size of purines. The importance of these residues in determining the –1 nearest neighbor preference was demonstrated earlier by mutagenesis experiments that implicated both D131 and Y132 of A3A (D314 and Y315 of A3B CD2 [60]) in determining the dinucleotide preference of 5′TC-3′, whereas the corresponding residues in A3G CD2 (D316 and D317) were shown to be responsible for its 5′-CC-3′ dinucleotide preference 17, 18, 60, 77, 78. A basic residue (R/K) at position 189 of α6-helix is conserved among catalytically active cytidine deaminase domains. In the A3A ligand bound structure, the basic residue at 189 stabilized the hydrogen bonding configuration of L7 with the –1 position base. Conservation of this bonding scheme underscores the importance of the –1 nucleotide interaction with APOBECs for the deamination reaction.

In the A3A co-crystal structures, the only observed interaction of the +1 base is that of a base-stack with H29. Base-stacks are typical of non-sequence-specific interactions with nucleic acid and may explain why among A3 proteins, the nucleotide preference at the +1 position is relatively low compared with that of the –1 position. Furthermore, the lack of specific base interactions with A3A beyond that of –1 through +1 may suggest why there is little to no target sequence preference among the majority of APOBECs outside of these two nearest neighbors of the targeted dC. The nucleotide bases at +2 and +3 positions were stacked on top of the +1 base (Figure 2A,B) and although they were proximal to L3, direct interactions of their bases or backbone were not observed. The lack of a substrate interaction with L3 was interesting because the conformation of unliganded L3 is highly plastic and titration of A3A with oligonucleotides induced conformational changes in L3 [71]. Thus, the role of L3 in substrate binding warrants further investigation. Parenthetically, though there are no crystal structures for A1, transcriptomic **CLIP-Seq** analysis and site-directed mutagenesis of *apoB* RNA substrates suggested lax sequence requirements for nucleotides in the +1 and –1 positions for RNA editing although A1 preferred to edit cytidines flanked by adenosine in these positions 2, 10, 79.

The overall U-shaped conformation of A3A ssDNA substrate has been corroborated by a **2D-**^**15**^**N-HSQC NMR** analysis of A3A (E72A) titrated with a series of ssDNA substrates that differ by a single nucleotide [80]. This ‘method of small changes’ allows for more precise assignment of chemical shift peaks and thus more informed modeling of bound ssDNA. However, in another sense, U-shaped conformation of substrate DNA complexed with A3A 76, 77, 80 was unexpected because this is completely different from prior predictions based on mutational and NMR studies 61, 71, 73, 81 (and reviewed in [1]).

The U-shape of ssDNA substrate is highly similar to the RNA substrate conformation in the TadA–tRNA complex [82]. Adenosine deaminases such as TadA share an evolutionarily conserved fold with APOBECs and one wonders if the A3A and TadA U- or hairpin-shaped nucleic acid substrate conformation is indeed common among polynucleotide-editing enzymes. In support of this contention, Holtz *et al*. [83] demonstrated that the loop region of stem–loop structures of ssDNA are preferred hotspots for APOBEC-mediated deamination of cytidine. And although AID and A3 cytidine deamination in context of RNA remains controversial, Sharma and Baysal [51] provided evidence suggesting that the substrate preference for RNA deamination by both A3A and A3G is found in the loop region of stem–loop structures. It remains likely that subtle differences in substrate binding conformation exist, even among other A3 proteins, that may determine RNA-editing capability. The role of RNA secondary structure in the mechanism for RNA-editing substrate recognition remains unresolved even for *bona fida* RNA-editing enzymes, like A1 84, 85.

### The Length of Loop 1 Affects the Catalytic Rate of Deamination for A3 Family

A3A and A3B (CD2) are nearly identical (∼90% sequence identity) and L1 accounts for almost half of the sequence differences between the two. The impact of L1 differences between A3A and A3B CD2 were evident in an NMR-based analysis of substrate binding and deaminase activity [72]. This structure demonstrated that the sequence and length of L1 in A3A contribute to the order-of-magnitude greater deaminase activity compared with native A3B, even though the proteins had nearly identical ssDNA binding affinities [72]. Substrates are occluded from the A3B active site due to a longer L1 of A3B CD2 compared with that in A3A [86] and the crystal structure of an unliganded A3B CD2 showed that the collapsed orientation of L1 (and L7) directly blocked substrate access to the active site [60]. The closed configuration of A3B appears to be unique among catalytically active APOBEC domains, in that substrate binding by A3B would require significant rearrangement of the surrounding residues, suggesting a structural constraint that may reduce the rate of A3B deamination.

## Nonspecific ssDNA Binding by Catalytic and Noncatalytic APOBEC Domains Regulate Substrate Recruitment

The structures of A3A and A3B–AL1 revealed important interactions for binding of ssDNA near the active site and for cytidine deamination in the context of preferred nearest neighbor sequence. However, additional substrate DNA interactions away from the active site have been proposed to regulate substrate–APOBEC interactions. The co-crystal structure of the catalytic domain of A3F (A3F CD2) with a 10 nt poly-dT ssDNA demonstrated that ssDNA can interact with APOBECs through residues distal to the active site in a non-sequence-specific manner. These interactions are of interest as they may support a regulatory function for capturing substrate nucleic acid and guiding it towards the active site or to an active site of an adjacent A3F catalytic domain in a homo-oligomeric complex [87] (Box 2). DNA binding to the catalytic domain of A3G (CD2) away from the active site has also been demonstrated by crosslinked mass spectrometry experiments [88]. In addition to such supplementary DNA binding sites on the catalytic domain, nucleic acid interactions with the noncatalytic CD1 of the dual-deaminase domain APOBECs also have a regulatory function for substrate interactions with the catalytically active CD2.

**Figure I:**
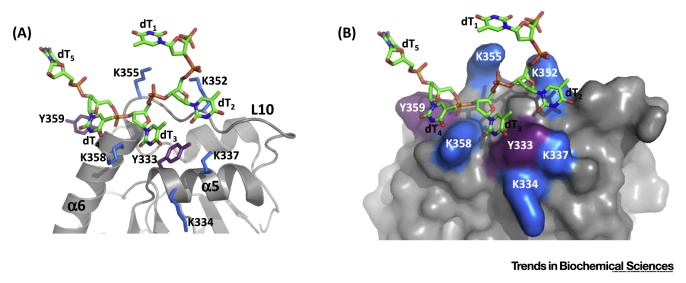
A Nonspecific DNA Binding Site on A3F Facilitates Substrate Catalysis at the Active Site. (A) The catalytic domain of A3F (CD2) with a poly-dT_10_ ssDNA (PDB 5W2 M, Table 1) co-crystallized with eight A3F (CD2) molecules and two strands of DNA in the asymmetric unit. A3F-based crystallographic interfaces are not robust enough to be true oligomeric interfaces, thus only one A3F (CD2) molecule is shown as a ribbon diagram. In this structure, the zinc ion is not coordinated canonically for a cytidine deaminase, but the CD fold is maintained and select secondary structural features are labeled accordingly. For clarity, only half (dT_1_ – dT_5_) of the poly-dT_10_ nucleic acid is shown in stick format with coloration the same as in Figure 1. (B) Close-up views of the five conserved lysines (blue sticks) and two conserved tyrosines (purple sticks) of α5-helix, L10, and α6-helix in ribbon diagram (top) and surface representation (bottom). Y333 pi-stacks with the dT_3_ base and Y359 stacks with the dT_4_ base. Extensive electrostatic interactions occur between lysines (K352, K355, and K358) and the phosphates of the DNA backbone. Both pi-stacking with bases and backbone electrostatic interactions are consistent with non-sequence-specific binding of nucleic acids.

Box 2 **Nonspecific ssDNA Binding by APOBEC3F May Guide Substrates to Active Site**

Unlike other APOBEC catalytic domains, A3F CD2 has a positively charged patch comprising five lysine residues (K334, 337, 352, 355, and 358) distal to the active site, located in α5 helix, loop 10, and α6-helix 65, 66, 87. The co-crystal structure showed these residues form an extensive network of electrostatic interactions with the negatively charged poly-dT backbone. Two tyrosine residues embedded in the positively charged surface patch (Y333 and 359) form hydrophobic pi-stacking interactions with several dT bases (Box 2, Figure IA and 1B). These interactions with ssDNA were typical of non-sequence-specific nucleic acid binding. Interestingly, the lysines were identified as critical for both ssDNA binding in **electrophoretic mobility shift assays** and for *in vitro* deamination of substrate ssDNA. Mutation of the hydrophobic tyrosines led to a greater reduction of deamination activity than of ssDNA binding. This suggested a model for the catalytic mechanism of A3F, in which ssDNA binds nonspecifically to the positive patch while specific ssDNA sequences may be selected and guided to the active site through hydrophobic interactions with the conserved tyrosines.

Alt-text: Box 2

### DNA Binding by Dual-Deaminase Domain APOBECs Regulates Catalytic Activity

In addition to the positively charged surface patch of A3F CD2, localized to L10 and α6-helix, CD2 L7 residue W310 and the equivalent tryptophan of CD1, W126, both contributed to ssDNA binding and catalytic activity of full-length A3F [89]. These data pinpointed relevant substrate interactions with A3F to three separate locations, spanning CD1 and CD2. Involvement of an A3F CD1 substrate binding interaction is consistent with the order of magnitude greater deamination rate observed for WT A3F compared with that of A3F CD2 alone [89].

The A3F CD2 positively charged surface patch is not conserved in other A3 members, but CD1 of A3G is nearly entirely positively charged and there is a growing body of evidence showing ssDNA binding by A3G CD1 is essential for the catalytic activity of CD2. First, A3G has been shown through mass spectroscopy (MS) of DNA crosslinked A3G to directly bind to DNA through at least three residues (Y181 and Y182 in CD1 domain and Y315 in CD2) [90]. Alanine substitutions at Y181 or Y182 reduced deaminase activity to half that of wild type (WT) A3G and A3G with an alanine substitution at Y315 had little or no activity [90]. Second, **atomic force microscopy** (AFM) experiments showed A3G may bind ssDNA in different modes depending on DNA length and each involved binding to both CD1 and CD2 [91]. Third, mutants of individual residues that mapped near the pseudo-catalytic site of A3G CD1 displayed significantly reduced deamination rates compared with WT A3G [64]. Fourth, like A3F, the CD2 domain of A3G (or A3B) alone had low or no ability to deaminate ssDNA substrate compared with their full-length counterparts, suggesting a regulatory role for the CD1 domain 73, 92, 93. The crystal structure of A3G CD1 (from rhesus macaque) with a poly-dT nucleic acid [69] revealed electron density for a small piece of DNA with a single dT nucleotide base bound in a catalytically incompetent mode within the pseudo-catalytic site of CD1. Similar to A3G, CD1 of A3B has two positively charged surface patches but it is unclear whether either patch binds to ssDNA to facilitate cytidine deamination by A3B CD2 [67]. The data suggested residues in A3G/A3F/A3B CD1 and CD2 removed from the active site that bind ssDNA. The requirement for these binding sites in guiding ssDNA substrates to the active site for catalytically productive interactions with APOBECs remains to be proven.

## AID Binds Structured ssDNA Substrates Using a Bifurcated Surface Groove

While A3A has been shown to bind linear ssDNA substrate at its active site, other APOBECs may have evolved to select substrates with different conformations in a manner that reflects specific functionality. Indeed, AID may have evolved to target structured DNA as substrate for cytidine deamination during CSR. The molecular mechanism of this deletional-recombination event requires double-stranded DNA breaks in switch region DNA to facilitate exchange of constant region genes. The current hypothesis is that sequences of G-repeats that are enriched in switch region DNA, form **G4 quadruplex** (G4) structures that may serve as a ‘guide’ in AID substrate recognition [94]. AID preferentially bound to and deaminated deoxycytidine in the context of G4-structured DNA substrates [95], rather than linear ssDNA substrates as for A3A and A3B–AL1 chimera 76, 77. AID preferentially deaminated deoxycytidines at the third position within the 5′ overhangs adjacent to G4 core structure with DNA substrates [95].

The crystal structure of a monomeric, catalytically active AID and a co-crystal structure of AID bound to a dCMP nucleotide was achieved following minimal mutation (F42E, H130A, R131E, F141Y, and Y145E) and short truncations of the N and C termini [95]. This structure revealed novel surface grooves that may serve as the molecular and structural foundation of AID substrate engagement and specific targeting of the class switch region DNA of the Ig gene by engaging a G4-quadruplexed substrate DNA (Figure 2C). The coordination of the dCMP base within the active site of AID is nearly identical to that of A3A [95]. Although AID residues of L1, L3, and L7 form a predicted substrate channel for neighboring nucleotides, as observed for A3A, the AID channel is not U-shaped. Instead, the AID surface near the active site had a straighter substrate ‘binding channel’ and an adjacent groove termed the ‘assistant patch’ (Figure 2C,D). Together, these two AID surface grooves form a bifurcated binding surface and are lined with basic residues (R/K), creating positively charged surfaces for binding to negatively charged DNA backbones. The grooves are separated near their point of convergence by negatively charged residues in Loop 7, termed the ‘separation wedge’ (Figure 2C,D). The basic residues of the grooves are highly conserved in AID of different species, but not among other APOBECs, suggesting that the deamination of ssDNA overhangs of G4 quadruplexes is a unique targeting mechanism of AID. However, a separation wedge was observed in structures of both T4 RNase H [96] and Cas9 [97] proteins that also recognize branched nucleic acids.

While the Pederson and Goodman labs also solved a structure of AID, their use of A3A loop sequences in an AID chimera protein for crystallization purposes prevented observation of bifurcated surface binding grooves [56]. Still, both AID structures do maintain the core cytidine deaminase fold, and together, this supports the importance of plasticity of this fold in retaining active site requirements of the family, despite each member evolving specific functions. These structures of AID 56, 95 and those of A3A revealed that despite the common catalytic mechanism for deamination, surface differences surrounding a common core fold may enable these enzymes to differentiate substrates.

## RNA Binding to A3G and A3B May Competitively and Allosterically Regulate Their Catalytic Activity

MS analysis of tryptic RNA- or DNA-crosslinked A3G peptides revealed that Y315 of A3G CD2 bound to ssRNA as well as ssDNA [90]. As discussed above, A3G Y315A mutants had little or no ability to bind RNA or DNA and were inefficient in assembling ribonucleoprotein (RNP) particles or hypermutating ssDNA [90]. RNA binding by A3G directly inhibited its ability to bind DNA [98]. The addition of RNA to an oligomeric complex of A3G assembled on ssDNA induced dissociation of A3G as a homodimer free of nucleic acid [88]. The data suggested a model wherein the mechanism for RNA inhibition of A3G ssDNA binding and catalytic deamination involves competitive RNA binding to Y315 within CD2 [90].

RNA binding to residues with CD1 of A3G has been demonstrated by site-directed mutagenesis [99] and through MS of A3G crosslinked to RNA [88]. These interactions have also been characterized as inducing homo-oligomerization of A3G as catalytically inactive high molecular weight RNP complexes 92, 98, 100, 101 (Box 3) and as being important for inhibition of endogenous retroelements (reviewed in [3]). Residues in CD1 are allosteric to those that coordinate ssDNA within the catalytic domain of CD2 and therefore RNA interactions with A3G CD1 may have a noncompetitive mechanism in modulating ssDNA deaminase activity.

Box 3 **Variation in Oligomerization of APOBEC Proteins Is Linked to Nucleic Acid Binding and Catalytic Function**

Many APOBECs utilize homo-oligomerization or complex formation to regulate activity and cellular distribution [3]. Dimeric A1 must form a complex with the A1 complementation factor (A1CF) or RBM47 [12] for docking with the mooring sequence 3′ of the editing site prior to deamination of the target C6666 in apoB mRNA [103]. Though the dimeric interface of A1 is not well established, a hydrophobic patch comprising α6-helix has been shown to modulate oligomerization. In contrast, the NMR solution structure of full-length (mouse) A2 predicted the protein is a monomer in solution [70], in agreement with the monomeric state observed by **fluorescence fluctuation spectroscopy** (FFS) of A2 in cells [117].

AID has been shown to form catalytically active homodimers 118, 119, but also to function as a monomer [120]. A recent model for CSR suggested that AID oligomerizes during G4 DNA binding, leading to accumulation of AID in IgS regions [95]. The tight association of AID with G4 DNA is believed to be responsible for AID localization on the ssDNA strand and the clustering of subsequent DNA mutations and double-stranded breaks requisite for such CSR. In contrast, mutational analysis of residues suspected to be involved in oligomerization suggested AID may function as a monomer in SHM, wherein the association of AID with IgV DNA was less stable and may have involved branched DNA but not the more complex G4-structured DNA as observed for CSR [95]. An RNaseA-sensitive AID dimer in complex with the heteronuclear ribonuclear protein (hnRNP) K [121] may be involved in DNA cleavage events associated with SHM [122]. HnRNP K was proposed to act as a cofactor (like that of A1CF for A1). AID dimers and monomers in complex with hnRNP L [121] have been proposed to be involved in DNA recombination events associated with CSR [122]. The structure-function requirement of these complexes for SHM and CSR and the role of RNA and RNA-binding proteins in these functions remain to be determined.

Oligomerization of some A3 proteins has been proposed to occur through either direct protein–protein interfaces or indirectly through nucleic acid mediated interactions, as described above for A3H. Accordingly, FFS has shown that A3B, A3D, A3F, A3G, and A3H form oligomers of varying size in cells while A3A and A3C, like A2, exist as monomers [117]. However, a crystal structure revealed A3A forms a homodimer mediated by a zinc-coordinated domain swap. While these data support a model of cooperative substrate binding and deamination [57], AFM showed A3A was predominantly monomeric when bound to ssDNA 123, 124, in agreement with the recent crystallographic 76, 77 and NMR-based analyses [80] of A3A with ssDNA. Dimerization of A3B through a catalytic domain interface has been reported [125] but several structural studies have suggested A3B oligomerization occurs through the noncatalytic domain 60, 72, 77, 86. It is important to keep in mind that purified A3B CD1 (much like A3G CD1) is prone to aggregation and has required extensive surface mutation to engineer a monomeric form for crystallization [67]. As such, one might anticipate that a dimeric interface within the current crystal structures would not be observable.

Dimeric interfaces for A3G have been suggested based on crystallographic contacts of catalytic CD2 structures and the noncatalytic CD1 structure, but little consensus exists as to the precise oligomeric state of catalytically active A3G or the mode of oligomerization (reviewed in [1]). Uncertainty persists because purified recombinant A3G can exist as a monomer, dimer, tetramer, and as higher order oligomers 93, 126, 127, 128, 129, and cellular-based FFS revealed A3G did indeed exist as a variety of oligomeric states [117]. Many reports have correlated A3G oligomerization with its catalytic activity 101, 126, 127, but evidence for catalytically active monomeric A3G has been reported [130] and a recent report showed that monomeric A3G becomes dimeric only after binding ssDNA [131]. Dimerization has been postulated to occur between either CD2 132, 133 domains or CD1 69, 93, 134, 135 domains. The recent crystal structure of A3G CD1 with a poly-dT nucleic acid revealed a potential dimeric interface comprising α6-helix and L7, which creates a large contiguous positively charged surface patch involving both CD1 moieties [69]. Notably, direct binding of ssDNA to A3G CD1 α6-helix through crosslinking was reported [88]. Poly-dT in the crystal structure of A3G was not modeled at this site [69].

An additional interface of great interest present in dual-deaminase domain A3s is that between CD1 and CD2. High-speed AFM imaging and molecular dynamic modeling were used to show monomeric A3G exists as a globular structure, in which both domains are closely associated, and alternately as a longer, dumbbell-shaped structure, in which domains are farther apart [136]. The domains are connected in cis by a short linker that forms a flexible coil, allowing the orientation between linked domains to change drastically on a short time-scale while preserving individual domain architecture. Though the functional implications of such an arrangement are not fully understood, the heterogeneity and dynamic nature of the interface has likely obfuscated numerous crystallization attempts of full-length A3G.

We speculate that the function of APOBEC oligomerization is to expand the molecular surface available for binding nucleic acid which in turn, enhances the ability to fine-tune regulatory mechanisms. However, the functional implications of a dynamic intermolecular interface for dual-deaminase A3s may further embellish the potential for regulation by allowing complicated binding modes involving multiple interactions that require timely reorientation of tethered domains. An additional area that will need resolution is the structure-function relationship that may arise from the interaction of CD1 and CD2 in trans between monomers in the oligomeric state of APOBECs that are mediated by protein–protein or nucleic acid–protein interactions.

Alt-text: Box 3

The noncatalytic A3B CD1 domain was shown to attenuate the native A3B deamination catalytic rate and ability of native A3B to invoke double-stranded breaks in DNA in cells [102]. The crystal structure of A3B CD1 revealed residues of L2, L4, and β5 strand create a patch of positive electrostatic potential (PDB 5TKM) [67]. A conservative mutational analysis of this patch showed that specific basic residues in this patch attenuate deamination of ssDNA through RNA binding, though it remains to be shown if this is a competitive binding site [67]. In contrast to the inhibitory mechanism when APOBEC oligomers are generated through RNP assembly, attenuation of A3B by RNA is less well understood, but it likely is functionally characteristic of the cellular regulation of APOBEC [103] (Box 3).

## Binding Structured RNA May Drive Virion Localization of Antiretroviral A3s

The four A3s (A3D, A3F, A3G, and A3H) that restrict HIV infection 54, 104 package with budding virions and, as such, exert their antiviral deaminase activity upon subsequent infection of a new cell (reviewed in [54]). Packaging of A3s in virions involves interactions with HIV nucleocapsid protein 105, 106, but also with cellular RNAs [107] and HIV genomic RNA [108] that have secondary structure features. A3G bound preferentially to the Alu secondary structure domain of cellular 7SL1 RNA *in vitro* but not to the linker or adjoining secondary structure known as the S domain [109]. Furthermore, A3G had greater than an order of magnitude higher affinity for the Alu domain of 7SL1 RNA compared with bulk cellular RNAs, regardless of whether they contain Alu repeats 107, 110, 111. A3G also showed binding selectivity for the conserved stem–loop structures 1 and 3 (SL1 and SL3) within HIV genomic RNA, and A3G interaction with SL1 enhanced the recovery of A3G with virions [108].

CLIP-Seq analysis of RNAs crosslinked to A3G in uninfected cells and infected cells, or recovered from virions, suggested that A3G RNA binding preferences changed upon infection. In uninfected cells, A3G was predominantly bound to random bulk mRNAs with a weak complement of **noncoding RNAs** (ncRNAs) 112, 113. Conversely, in HIV virions, A3G was predominantly bound to 7SL1 RNA and HIV genomic RNA bound to A3G in HIV virions [113], as well as a lower complement of other ncRNAs 112, 113. Thus, A3G interactions with RNA are context-dependent and localization of A3G to virions is likely dependent upon RNAs with unique secondary structure. The extent to which other anti-HIV A3s depend on RNA secondary structure remains to be determined.

Interestingly, CLIP-Seq analysis of genomic RNA from HIV particles revealed that the most frequently bound sites by human A3H are predicted to form duplexes of at least seven nucleotides in length [113]. A3H of either a pig-tailed macaque natural polymorphic variant [114] or of human haplotype II [115] co-crystallized with cellular-derived A-form RNA duplexes. The mixed sequence duplexes were comprised of nine nucleotides per strand, of which seven were paired (Figure 2E). The RNA binding site on A3H is a large basic, positively charged surface patch comprising several arginine residues of α6-helix that hydrogen bond with the RNA phosphate backbone of both strands; it is distinct from that of the deamination active site (Figure 2E inset). Further stabilization was visualized by base stacking with tryptophan of the A3H-specific RNA binding motif ^110^RLYYHW^115^ of L7 and a tyrosine within the four residue-insertion sequence ^22^PYYP^25^ specific to A3H, located in L1 [116] (Figure 2E inset). Although the RNA duplex is not bound to the A3H active site, purified A3H–RNA complexes are not catalytically active unless treated with RNase A 59, 114, 115. The crystal structure of human apoA3H (no RNA bound) revealed the conformation of L1 and L7 of unbound A3H differ from that of RNA-bound A3H [59], suggesting that RNA must be released in order for the active site to have the conformational flexibility to bind ssDNA substrate (Figure 2F).

Residues of the human A3H that directly interact with the RNA duplex were shown to be required for RNA binding, RNA-mediated oligomerization, and HIV virion packaging and restriction [115]. In addition, residues of the basic patch were shown to be the sole determinants of RNA-mediated inhibition of ssDNA deamination activity [115].

These crystallographic models are the first of an APOBEC complexed with RNA and provide a basis for understanding RNA-based virion encapsidation and potentially RNA-based inhibition of ssDNA deamination. However, it is unlikely that the other antiretroviral A3s utilize this specific mode of RNA binding for virion encapsidation because the L1 insert sequence critical for RNA duplex binding is unique to A3H. Although other A3s have highly basic surfaces, the other RNA binding domains (putatively CD1) of A3D, F, and G do not have the same basic patch on α6-helix. A notable exception is A3C, whose α6-helix is highly homologous to that of A3H [115]. Still, the abundance of positively charged surface patches on antiretroviral A3s, the enrichment of highly structured ncRNAs (including 7SL RNA) discovered within A3G CLIP-Seq data 112, 113, and the preference of A3G for binding SL1 and SL3 HIV gRNA [108] does suggests that binding RNA secondary structural elements for virion encapsidation may be common among antiretroviral A3s. Additional analysis of RNA secondary structure requirements for APOBEC binding to nucleic acids is needed.

## Concluding Remarks

Our structural based understanding of APOBEC substrate selection and binding, catalytic deamination, regulation, and subcellular localization may still be in its infancy, yet the field has made significant progress in elucidating the structural underpinnings of APOBEC interactions with nucleic acid. The interaction with the cytidine base in the active site pocket appears similar for co-crystal structures of A3A and AID and although they are likely representative of active site interactions for the rest of the family, differences among APOBEC surfaces both near to and far from the active site point to functional differences among members. These differences are important to understand as they may represent the underlying mechanism for diversification of APOBEC function. The substrate binding channel for A3A is U-shaped and accepts linear ssDNA, while that of AID is straighter and the presence of an additional ‘assistant patch’ attracts G4-structured DNA. Whether the U-shape of the A3A substrate is common among substrates for other APOBECs that act upon linear substrates should be a focus of future structural studies (see Outstanding Questions). For APOBECs that deaminate cytidine in the context of RNA (A1, A3A, and A3G), an equally enticing question is whether RNA secondary structure contributes to the selection of target cytidines at the active site, and for A3A and A3G, whether RNA and DNA substrates have similar configuration.

The presence of positively charged surface patches on the catalytic domain of A3F distal to the active site as well as on noncatalytic domains of A3B and A3G present expanded opportunities for nonspecific sequences to bind and modulate catalytic activity. The identification of RNA-binding sites on both A3G domains adds a layer of complexity to the RNA-mediated inhibition of substrate binding and catalytic activity as well as to RNA mediated formation of high molecular weight RNP complexes. RNA binding to the noncatalytic domain of A3B suggests a similar mechanism of modulation of its catalytic activity. However, the mechanism for RNA regulation of catalytic activity is not known. Likewise, it is unclear if multiple nucleic acids can be bound simultaneously to APOBECs at different sites. To fully appreciate the physiological roles of APOBEC proteins, these questions will need to be addressed.

It is also apparent that structured nucleic acids play a key role both in regulating catalytic activity, as shown by the AID preference for G4-quadruplexed DNA substrates, and in virion localization, as shown by A3G and A3H preference for viral-associated structured RNAs. Yet, it is still unclear if, and how, RNA sequence or secondary structure impacts regulation of catalytic activity of APOBECs through binding to regulatory sites on either catalytic or noncatalytic domains.

Oligomerization of APOBECs is an essential aspect of APOBEC molecular function. Of utmost importance is the question of how homo-dimers or homo-tetramers modulate function through nucleic acid binding at either the catalytic site(s), nonspecific sequence binding sites, or RNA-binding sites. A3s with tandem deaminase domains introduce another layer of complexity for understanding how multiple deaminase domains affects protein oligomerization, nucleic acid binding, and deoxycytidine site selection along a stand of ssDNA. The potential of an ill-defined and dynamic interface between tandem cytidine deaminase domains (as observed for A3G) makes this question exceedingly difficult to address but critical to resolve.

It is apparent that RNA (and DNA) binding regulates catalytic activity and localization of numerous APOBECs but a role for regulation by a protein cofactor(s) has only been established for A1 (Box 1). While some A3s interact with HIV nucleocapsid protein to drive virion encapsidation, little else is known about protein–protein interactions that may affect APOBEC activity or localization. Likewise, the mechanisms that prohibit cancer-causing genomic editing by nuclear-localized APOBECs are also not fully understood.

Further structural studies are needed to understand the varying functional roles of APOBEC proteins in health and disease and to determine how specific and nonspecific RNA and ssDNA binding of these proteins may itself affect cellular function(s). This too is fundamental to our appreciation of the physiological significance of APOBEC-mediated RNA and genome editing and to our ability to technologically exploit their value in gene and cell engineering or drug development.

Outstanding Questions Is the binding mechanism of A3A to linear ssDNA substrate common among other APOBECs that bind linear ssDNA? Is AID the only APOBEC that binds a structured or branched ssDNA?

How does the overall sequence or secondary structure context of ssDNA or RNA substrate affect APOBEC binding?

How do the additional positively charged nucleic acid binding surfaces distal to the active site affect substrate binding and APOBEC function? Can multiple substrate or nonsubstrate nucleic acids bind simultaneously to multiple sites on an APOBEC monomer?

Do APOBECs use a common structural configuration within the active site to deaminate both RNA and DNA?

What are the regulatory mechanisms that prevent off-target APOBEC-mediated genomic mutagenesis? Is A1 the only APOBEC that requires a cofactor for nucleic acid binding and editing site selectivity.?

What are the oligomeric states of catalytically competent and catalytically inactive APOBEC proteins?

Is the dynamic orientation between CD1 and CD2 seen with A3G common among the other A3 proteins with dual-deaminase domains (A3B, A3D, and A3F)? Does the orientation between domains become fixed upon substrate binding?

Aside from RNA-binding, are there other regulatory mechanisms determining structural and functional characteristics of APOBEC proteins?

What is the structural mechanism by which RNA regulates the antiretroviral activity of different A3 proteins?

## Glossary

**2D-^15^N-HSQC NMR**: two-dimensional heteronuclear single quantum coherence nuclear magnetic resonance experiment that determines the coupling of the hydrogens (^1^H) and nitrogen isotope, ^15^N, of a protein that has typically been labeled with the isotope. The coupling is used to determine the chemical shift of each amide, which can reveal information about the protein structure.
**Atomic force microscopy**: a type of high-resolution scanning probe microscopy, employing a cantilevered tip that raster-scans over a biological sample preparation on a microscopic stage. Changes in height of the tip during sample scans are recorded and processed to generate an image of all samples on the stage.
**CLIP-Seq**: a method that combines the high-throughput sequencing of RNA isolated by UV-induced crosslinking immunoprecipitation to identify RNA sequences that serve as binding sites for protein.
**Co-crystal structure**: a structural model of a protein with a bound ligand (e.g., nucleic acid or small molecule).
**Cytidine to uridine deamination**: the modification of a cytidine base to a uridine base through enzymatic removal of the exocyclic NH_2_ group at the C4 position of cytidine. The reaction is catalyzed by a cytidine deaminase that uses a coordinated zinc ion to activate a water molecule for nucleophilic attack at the C4 position, while a conserved glutamic acid shuttles a proton for formation of the NH_3_ leaving group.
**Electrophoretic mobility shift assays**: EMSA is a technique to monitor the formation of protein–nucleic acid complexes with a nondenaturing polyacrylamide or agarose gel. The mobility of a protein-bound nucleic acid in an electrophoretic gel is retarded compared with that of the free nucleic acid and the band migration pattern can be used to assess size, conformation, and binding kinetics of protein–RNA complexes.
**Fluorescence fluctuation spectroscopy**: a technique that measures the change of fluorescence intensity over short time periods in a defined volume of a living cell that has expressed a fluorescently tagged protein. Analysis of brightness or the autocorrelation of intensity changes yields information about the concentration and stoichiometry of the fluorescent protein.
**G4 quadruplex**: nucleic acid sequences that are rich in guanine (G) form secondary structures of planar guanine tetrads in which four guanines associate through Hoogsteen hydrogen bonding. A stack of guanine tetrads is known as a G4 quadruplex. These structures occur in transcriptional regulatory regions and in telomeres and may serve to recruit small molecules or proteins for a variety of functions.
**Hypermutation**: the occurrence of numerous nucleotide editing events on a single strand of DNA or RNA.
**Long interspersed nuclear elements (LINE)**: a type of retrotransposon that does not encode long terminal repeat (LTR) sequences (non-LTR), but whose genes do code for all functions necessary for replication (reverse transcriptase, endonuclease, and an ability to form ribonucleoprotein complexes) and as such are deemed autonomous.
**Noncoding RNAs**: transcribed RNAs that are not used for translation of protein, but instead serve in a wide variety of cellular functions, including roles in the molecular machinery for translation (transfer RNA, ribosomal RNA, and those associated with the signal recognition particle), RNA-splicing, DNA replication, and regulation of gene expression, genome defense and even as hormones.
**Retrotransposition**: the replicative mode of a retrotransposon, a parasitic genetic element in eukaryotic genomes, that involves transcription to an RNA intermediate and reverse transcription back to DNA, followed by integration back into a host genome at specific sites.
**Short interspersed nuclear elements (SINE)**: a non-LTR type of retrotransposon, similar to LINE, but does not code for all functions necessary for replication and co-opt those of LINE machinery instead and are thus nonautonomous.
**Substrate and nonsubstrate nucleic acids**: an APOBEC substrate nucleic acid is one that has a (deoxy)cytidine within a context capable of being recognized and edited by an appropriate APOBEC. A nonsubstrate nucleic acid lacks a (deoxy)cytidine or the correct context for editing. A nonsubstrate nucleic acid may still be bound by and regulate an APOBEC protein.
